# Clinical characteristics associated with COVID-19 severity in California

**DOI:** 10.1017/cts.2020.40

**Published:** 2020-04-16

**Authors:** Samuel J. S. Rubin, Samuel R. Falkson, Nicholas R. Degner, Catherine Blish

**Affiliations:** 1 Department of Medicine, Stanford University School of Medicine, Stanford, CA, USA; 2 Chan-Zuckerberg Biohub, San Francisco, CA, USA

**Keywords:** COVID-19, SARS-CoV-2, oxygen saturation, hypertension, acute respiratory distress syndrome (ARDS), severe acute respiratory syndrome (SARS)

## Abstract

Given the rapidly progressing coronavirus disease 2019 (COVID-19) pandemic, this report on a US cohort of 54 COVID-19 patients from Stanford Hospital and data regarding risk factors for severe disease obtained at initial clinical presentation is highly important and immediately clinically relevant. We identified low presenting oxygen saturation as predictive of severe disease outcomes, such as diagnosis of pneumonia, acute respiratory distress syndrome, and admission to the intensive care unit, and also replicated data from China suggesting an association between hypertension and disease severity. Clinicians will benefit by tools to rapidly risk stratify patients at presentation by likelihood of progression to severe disease.

## Introduction

The coronavirus disease 2019 (COVID-19) pandemic caused by severe acute respiratory syndrome coronavirus 2 (SARS-CoV-2) poses a global threat. Disease severity varies widely from mild (81%), requiring hospitalization (12%), to death (1.8–3.4%) [1].

The growing number of US COVID-19 cases is expected to tax the capacity of health care delivery systems. Analysis of clinical characteristics at time of patient presentation associated with disease severity is immediately useful. Recognizing the rapid utility of such data, we evaluated clinical characteristics and disease course of patients with confirmed COVID-19 at Stanford Hospital.

## Methods

With approval of the Stanford Institutional Review Board, patient charts were analyzed if they were diagnosed with COVID-19 by reverse transcription polymerase chain reaction, received care at Stanford Hospital by March 16, 2020, and had past medical history documentation. Statistical analyses were conducted in Microsoft Excel and R.

## Results

Of 54 patients analyzed, the median age was 53.5 years (interquartile range, 32.75; range, 20–91), 53.7% were male, 18 were inpatients, and 36 were outpatients (Table 1). Based on chart documentation of past medical history, 14 had hypertension, 13 had hyperlipidemia, and 7 had diabetes (6 type 2, 1 type 1).

**Table 1.** Clinical characteristics of 54 patients with COVID-19 in California

	Female (*N* = 25)	Male (*N* = 29)	*P*-value
Baseline characteristics – no. of patients (% of all patients)			Fisher’s exact
Race/ethnicity			
White, non-Hispanic/non-Latino	8 (14.8)	16 (29.6)	0.220
Asian, non-Hispanic/non-Latino	6 (11.1)	3 (5.6)	0.479
Other, non-Hispanic/non-Latino	5 (9.3)	5 (9.3)	1.000
Other, Hispanic/Latino	1 (1.9)	2 (3.7)	1.000
Native Hawaiian or other Pacific Islander, non-Hispanic/Non-Latino	3 (5.6)	0 (0)	0.239
Unknown	2 (3.7)	3 (5.6)	1.000
Prediagnosis comorbidities			
Asthma	3 (5.6)	0 (0)	0.239
Atrial fibrillation	1 (1.9)	2 (3.7)	1.000
Autoimmune disease	3 (5.6)	2 (3.7)	1.000
Chronic kidney disease	0 (0)	2 (3.7)	0.492
Coronary artery disease	0 (0)	2 (3.7)	0.492
Depression	3 (5.6)	2 (3.7)	1.000
Diabetes, pre	0 (0)	2 (3.7)	0.492
Diabetes, type 1	1 (1.9)	0 (0)	1.000
Diabetes, type 2	3 (5.6)	3 (5.6)	1.000
End-stage renal disease	1 (1.9)	0 (0)	1.000
Fatty liver disease	1 (1.9)	1 (1.9)	1.000
Hepatitis B virus infection	0 (0)	1 (1.9)	1.000
Hyperlipidemia	5 (9.3)	8 (14.8)	0.545
Hypertension	7 (13.0)	7 (13.0)	1.000
Ischemic cardiomyopathy	0 (0)	1 (1.9)	1.000
No previous medical condition	9 (16.7)	12 (22.2)	0.616
Obstructive sleep apnea	3 (5.6)	1 (1.9)	0.612
Paroxymal atrial flutter	0 (0)	1 (1.9)	1.000
Medications			
Angiotensin-converting enzyme inhibitors	3 (5.6)	1 (1.9)	0.612
Angiotensin II receptor blockers	1 (1.9)	4 (7.4)	0.356
Statins	4 (7.4)	5 (9.3)	1.000
History and presentation findings – mean values (SD, *n*)			Student’s *t*
Age (years)	53.6 (19.1, 25)	53.0 (20.0, 29)	0.916
Systolic blood pressure (mmHg)	124.3 (19.8, 16)	131.3 (19.5, 21)	0.292
Respiratory rate (bpm)	18.2 (2.4, 17)	20.7 (5.2, 18)	0.074
Temperature (ºC)	37.3 (0.7, 19)	37.4 (0.9, 23)	0.970
Oxygen saturation (%)	97.1 (3.3, 20)	95.7 (6.2, 24)	0.389
Laboratory findings – mean values (SD, *n*)			Student’s *t*
Serum potassium (mmol/L)	4.2 (0.7, 9)	3.9 (0.5, 16)	0.300
Serum sodium (mmol/L)	135.1 (2.7, 9)	135.9 (5.0, 16)	0.654
Serum calcium (mg/dL)	9.0 (0.3, 9)	8.9 (0.5, 14)	0.731
Serum bicarbonate (mmol/L)	24.2 (4.0, 9)	25.1 (2.5, 16)	0.527
Platelets (×1e3/µL)	214.1 (64.8, 10)	189.7 (57.5, 15)	0.334
Red blood cells (×1e6/µL)	4.6 (0.4, 9)	4.8 (0.5, 14)	**0.012**
White blood cells (×1e3/µL)	6.0 (2.1, 10)	7.0 (2.6, 15)	0.324
Absolute lymphocyte (×1e3/µL)	1.6 (1.0, 8)	1.0 (0.5, 14)	0.062
Absolute neutrophil (×1e3/µL)	4.5 (1.5, 9)	5.1 (2.8, 14)	0.567
Creatinine (mg/dL)	1.8 (3.0, 9)	1.0 (0.2, 14)	0.350
Asparate aminotransferase (U/L)	73.4 (61.8, 9)	45.1 (19.5, 14)	0.122
Alanine aminotransferase (U/L)	69.6 (65.2, 9)	43.9 (25.8, 13)	0.212
Clinical progression – no. of patients (% of all patients)			Fisher’s exact
Recommended further in-hospital care	7 (13.0)	11 (20.4)	0.587
Hospitalized	6 (11.1)	10 (18.5)	0.410
Admitted to intensive care unit	3 (5.6)	3 (5.6)	1.000
Evidence of co-infections			
Viral	4 (7.4)	1 (1.9)	0.356
Bacterial	0 (0)	1 (1.9)	1.000
Diagnosis of pneumonia	7 (13.0)	11 (20.4)	0.587
Progression to acute respiratory distress syndrome	1 (1.9)	3 (5.6)	0.612

*N*, total patient number in category; *n*, patients in category with data available; SD, standard deviation.

The statistically significant values are in bold.

Consistent with previous COVID-19 reports, the most common prediagnosis comorbidity in 24 patients (25.9%) was hypertension; this rate does not significantly differ from the US prevalence (Fisher’s exact test, *P* = 0.671) [2,3]. Angiotensin-converting enzyme inhibitor (ACE-I) or angiotensin II receptor blocker (ARB) use in nine patients (16.7%) was also not significantly different from the US prevalence (Fisher’s exact test, *P* = 1.000) [4].

In univariate analysis, age, lower oxygen saturation at initial examination, and hypertension were significantly associated with recommendation for further hospital care, lower oxygen saturation was associated with admission to intensive care unit (ICU), age and lower oxygen saturation were associated with diagnosis of pneumonia, and lower oxygen saturation and hypertension were associated with progression to acute respiratory distress syndrome (ARDS) (Table 2). Use of ACE-I or ARB was not significantly associated with recommendation for further hospital care, admission to ICU, diagnosis of pneumonia, or progression to ARDS. When analyzed by logistic regression to control for age, the only factor independently significantly associated with recommendation for further in-hosptial care, diagnosis of pneumonia, and progression to ARDS was initial oxygen saturation measurement as a continuous variable.

**Table 2.** Correlates of clinical progression

	Univariate	Multivariate
	*P*-value	*P*-value
Recommended further in-hospital care		
Age^*^	**0.003**	0.926
Oxygen saturation at history and presentation^*^	**4.730 E-04**	**0.010**
History of hypertension^†^	**0.024**	0.138
Use of ACE-I^†^	1.000	0.339
Use of ARB^†^	0.057	0.239
Admission to intensive care unit		
Age^*^	0.204	0.879
Oxygen saturation at history and presentation^*^	**6.667 E-04**	0.067
History of hypertension^†^	0.173	0.268
Use of ACE-I^†^	1.000	0.995
Use of ARB^†^	0.459	0.433
Diagnosis of pneumonia		
Age^*^	**0.027**	0.862
Oxygen saturation at history and presentation^*^	**0.001**	**0.026**
History of hypertension^†^	0.512	0.186
Use of ACE-I^†^	0.289	0.994
Use of ARB^†^	1.000	0.075
Progression to acute respiratory distress syndrome		
Age^*^	0.151	0.578
Oxygen saturation at history and presentation^*^	**3.476 E-05**	**0.029**
History of hypertension^†^	**0.049**	0.094
Use of ACE-I^†^	1.000	0.997
Use of ARB^†^	0.330	0.467

ACE-I, angiotensin-converting enzyme inhibitors; ARB, angiotensin II receptor blockers.

The statistically significant values are in bold.

* Two-tailed homoscedastic Student’s *t*-test for univariate analysis.

† Fisher’s exact test for univariate analysis.

## Discussion

Clinical characteristics of US COVID-19 patients and factors from initial presentation that associate with disease severity were identified. Lower oxygen saturation at presentation was independently significantly associated with measures of disease severity and thus may serve as a useful indicator of potential disease progression. Additionally, history of hypertension predisposed patients to worse outcomes such as ARDS, although not independently of age, consistent with previous reports [2].

Several factors, including age-related changes in the immune system and other physiological processes, could contribute to COVID-19 disease severity. While hypertension is associated with COVID-19 morbidity, it is not independent of age; thus further study is needed to elucidate the extent to which hypertension and/or dysregulation of the renin–angiotensin–aldosterone system (RAAS) contribute to COVID-19 pathogenesis. The idea that RAAS dysregulation may influence disease progression warrants further exploration into the usefulness of ACE-I and/or ARB therapies in COVID-19 management.

The potential role of the RAAS system in COVID-19 pathogenesis stems from mounting sequence and structural evidence indicating entry of SARS-CoV-2 via interaction of its spike protein with its human host receptor ACE2 [5]. It is difficult to predict the effects of RAAS blockade in treatment of COVID-19, as it may increase expression of ACE2, with known anti-inflammatory and pulmonary protective properties, yet simultaneously promote viral entry [6]. However, RAAS blockade may also increase soluble ACE2, which could serve as a decoy receptor protecting against viral entry.

In our study, history of ACE-I or ARB use did not affect diagnosis rate or predispose patients to worse disease outcomes. However, our study is underpowered to draw definitive conclusions from such negative data. We hope these initial findings motivate larger studies intended to characterize RAAS blockade in the COVID-19 setting.

Limitations of this study include lack of some data on disease progression due to retrospective design and potential selection bias of patients with severe disease more likely to have SARS-CoV-2 testing and extensive charting. However, these US data are immediately clinically relevant given the rapidly evolving pandemic and will help clinicians identify and treat patients most at risk of severe disease.
